# Allometric models and aboveground biomass stocks of a West African Sudan Savannah watershed in Benin

**DOI:** 10.1186/s13021-016-0058-5

**Published:** 2016-08-17

**Authors:** Adéyèmi Chabi, Sven Lautenbach, Vincent Oladokoun Agnila Orekan, Nicholas Kyei-Baffour

**Affiliations:** 1 Department of Civil Engineering, Kwame Nkrumah University of Science and Technology, Kumasi, Ghana; 2 Faculty of Agriculture, Institute of Geodesy and Geo-Information, University of Bonn, Nussallee 1, 53115 Bonn, Germany; 3 Department of Geography, University of Abomey-Calavi, BP 677, Abomey-Calavi, Benin; 4 Department of Agricultural Engineering, The College of Engineering, Kwame Nkrumah University of Science and Technology, Kumasi, Ghana; 5 West African Science Service Centre on Climate Change and Adapted Land Use (WASCAL), Accra, Ghana

**Keywords:** Allometric models, Aboveground biomass stocks, West African Sudan Savannah watershed, Non-destructive method, Biomass density, Benin

## Abstract

**Background:**

The estimation of forest biomass changes due to land-use change is of significant importance for estimates of the global carbon budget. The accuracy of biomass density maps depends on the availability of reliable allometric models used in combination with data derived from satellites images and forest inventory data. To reduce the uncertainty in estimates of carbon emissions resulting from deforestation and forest degradation, better information on allometric equations and the spatial distribution of aboveground biomass stocks in each land use/land cover (LULC) class is needed for the different ecological zones. Such information has been sparse for the West African Sudan Savannah zone. This paper provides new data and results for this important zone. The analysis combines satellite images and locally derived allometric models based on non-destructive measurements to estimate aboveground biomass stocks at the watershed level in the Sudan Savannah zone in Benin.

**Results:**

We compared three types of empirically fitted allometric models of varying model complexity with respect to the number of input parameters that are easy to measure at the ground: model type I based only on the diameter at breast height (DBH), type II which used DBH and tree height and model type III which used DBH, tree height and wood density as predictors. While for most LULC classes model III outperformed the other models even the simple model I showed a good performance. The estimated mean dry biomass density values and attached standard error for the different LULC class were 3.28 ± 0.31 (for cropland and fallow), 3.62 ± 0.36 (for Savanna grassland), 4.86 ± 1.03 (for Settlements), 14.05 ± 0.72 (for Shrub savanna), 45.29 ± 2.51 (for Savanna Woodland), 46.06 ± 14.40 (for Agroforestry), 94.58 ± 4.98 (for riparian forest and woodland), 162 ± 64.88 (for *Tectona grandis* plantations), 179.62 ± 57.61 (for *Azadirachta indica* plantations), 25.17 ± 7.46 (for *Gmelina arborea* plantations), to 204.92 ± 57.69 (for *Eucalyptus grandis* plantations) Mg ha^−1^. The higher uncertainty of agroforestry system and plantations is due to the variance in age which affects biomass stocks.

**Conclusion:**

The results from this study help to close the existing knowledge gap with respect to biomass allometric models at the watershed level and the estimation of aboveground biomass stocks in each LULC in the Sudan Savannah in West Africa. The use of model type I, which relies only on the easy to measure DBH, seems justified since it performed almost as good as the more complex model types II and III. The work provided useful data on wood density of the main species of the Sudan Savannah zone, the related local derived biomass expansion factor and the biomass density in each LULC class that would be an indispensable information tool for carbon accounting programme related to the implementation of the Kyoto Protocol and REDD+ (reducing emissions from deforestation and forest degradation, and forests conservation, sustainable management of forests, and enhancement of forest carbon stocks) initiatives.

**Electronic supplementary material:**

The online version of this article (doi:10.1186/s13021-016-0058-5) contains supplementary material, which is available to authorized users.

## Background

The sources and sinks of carbon from land use and land cover change (LULCC) are significant elements in the global carbon budget [1]. Current challenges of forest management are related to verifiable, reliable, accurate and cost-effective methods to adequately document forest resources dynamics [2]. The accuracy of biomass density maps depends on the availability of reliable allometric models to infer aboveground biomass (AGB) of trees from tree census data [3]. Large uncertainties in emission estimates arise from inadequate data on the biomass density of forests and the regional rates of deforestation [1, 4]. These uncertainties compromise the estimation of terrestrial carbon emissions [5–8] and required knowledge on biomass stocks.

A number of comprehensive allometric models for biomass estimation have been developed for the major tree species in Europe, America and Asia [3, 9–22]. In sub-Saharan Africa and especially West-African countries, most of the estimation of the total carbon stocks has also used allometric models together with forest inventory data [22–35]. The majority of studies so far have focused on forest ecosystems, specific tree species or plantations for the estimation of AGB and carbon stocks [3, 23, 25, 26, 28, 31, 32, 36–46]. Very few studies have dealt with the estimation of AGB in the agricultural landscapes [35].

Attempts to estimate AGB at the watershed level requires typically satellite images derived LULC information as well as allometric models from each LULC class. The data for allometric models for estimating biomass in woody vegetation comes either from destructive or from non-destructive methods. Destructive methods are based on the harvesting of the living trees together with measurements of diameter at breast height (DBH) or stem girth and total height as well as the dry mass of stem, foliage and branches. The collected variables are then used as input for estimating tree volume and biomass for selected trees species [22, 30, 37, 45, 47]. According to Djomo et al. [25], the application of destructive methods is labour intensive and time consuming. This method is therefore restricted to small trees at small scales [38, 48]. Additionally, harvesting trees requires in general special authorization which is often not easy to acquire especially when the study region involves protected areas.

Recent assessments have switched to the use of non-destructive methods [42, 49–55]. The tools and approaches used thereby vary considerably between regions. A biomass expansion factor (BEF) which expresses the relationship between stem biomass and the total biomass of a single tree species as well as information on wood density of the involved tree species are the keys variables used by allometric models to assess total biomass of living trees. If shape characteristics are included in the estimation of the BEF the approach is similar to the volume based approach in which information on height and diameter of a tree are used together with species specific shape and wood density factors [82]. The importance of wood density for estimating forest biomass and greenhouse-gas emissions from LULCC has been stressed by Nogueira et al. [54]. A variety of different approaches has been applied in case studies worldwide: Montes et al. [53] e.g. estimated the biomass of thuriferous juniper woodland in Morocco based on component volumes estimated from two orthogonal-view photographs and the density of each component. This approach is not well suited to estimate biomass in natural environments, especially when the environment is degraded by human use and wood supply for the local populations is at stake. Lehtonen et al. [52] developed expansion factors conditional on stand age and dominant tree species to estimate total biomass of pine trees in Norway. Flombaum and Sala [50] presented an approach for the calibration of a fast non-destructive method to estimate aboveground plant biomass by double-sampling vegetation cover and AGB in the Patagonian steppe. The author fitted linear regression models to describe the relationship between vegetation cover and biomass for the dominant species and life forms. Tackenberg [55] presented a non-destructive method based on scaled digital images analysis of the plants silhouettes, addressing not only aboveground fresh biomass and oven-dried biomass, but also vertical biomass distribution as well as dry matter content and growth rates. The method used by Tackenberg [55] is time and cost effective compared with destructive measurements, especially if development or growth rates are to be measured repeatedly. Another branch of approaches aims at identifying wood volumes by remote sensing approaches [83–86]—however for relating volume estimates with biomass information on wood density for the relevant species is necessary which is missing for many natural and semi-natural tree species in the tropics and sub-tropics.

Two problems hinder the transfer of the currently used non-destructive methods in the West-African context. First, BEFs are not available for most relevant local tree species and most devices used in other regions of the world are not suitable. In the southern part of the Republic of Benin, Guendehou et al. [42] assessed stem biomass based on stem volume and wood density for selected tropical tree species using an increment borer as the device of stem wood sample extraction. Unfortunately, the obtained BEF could not be applied in the context of the present study since the study was undertaken under the tropical conditions in West Africa which are different from the conditions in the case study region. The work by Guendehou et al. [42] therefore needs to be expanded to reflect conditions and tree species in different land use systems to allow a more precise estimation of the relevance of African trees for biomass and carbon stocks.

The goal of this paper was to accurately estimate AGB stocks at the watershed level in the Sudan Savannah zone using satellite images derived LULC data and adjusted allometric models based on data from non-destructive method.

## Methods

**Fig. 1:**
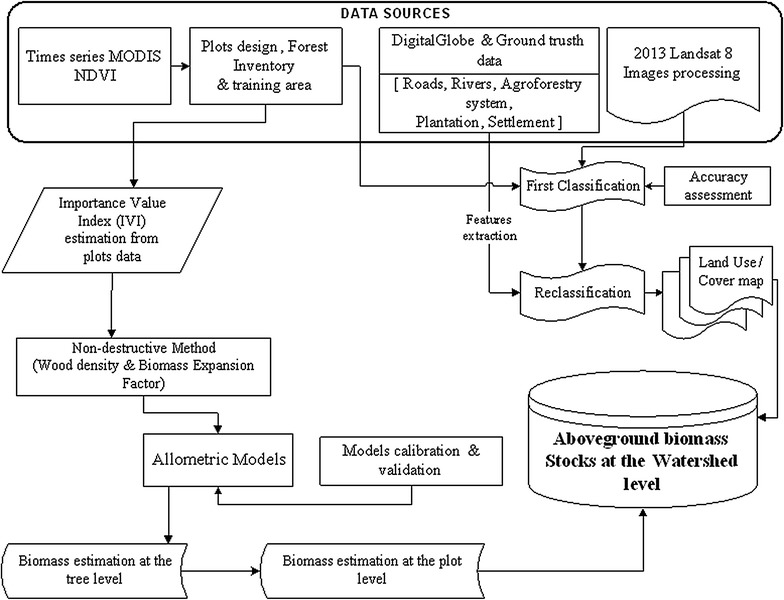
Flowchart showing the approach for the estimation of aboveground biomass at the watershed scale

The AGB estimation at the watershed scale was based on the following steps (Fig. 1):

1. satellite images analysis and LULC classification,
2. forest inventory in each identified LULC class of the watershed,
3. trees communities analysis and identification of the main species of the watershed based on importance value index (IVI),
4. estimation of basic wood density and BEF model of selected species,
5. assessment of non-destructive method to the destructive one based on available data,
6. development of allometric models using DBH, tree height and basic wood density of main trees species,
7. calculation of biomass data at the tree and the plot level using the best allometric equations of each LUCa and extrapolation at the watershed level,
8. mapping the biomass density using ArcGIS 10.2.1 software.

### Site location

**Fig. 2:**
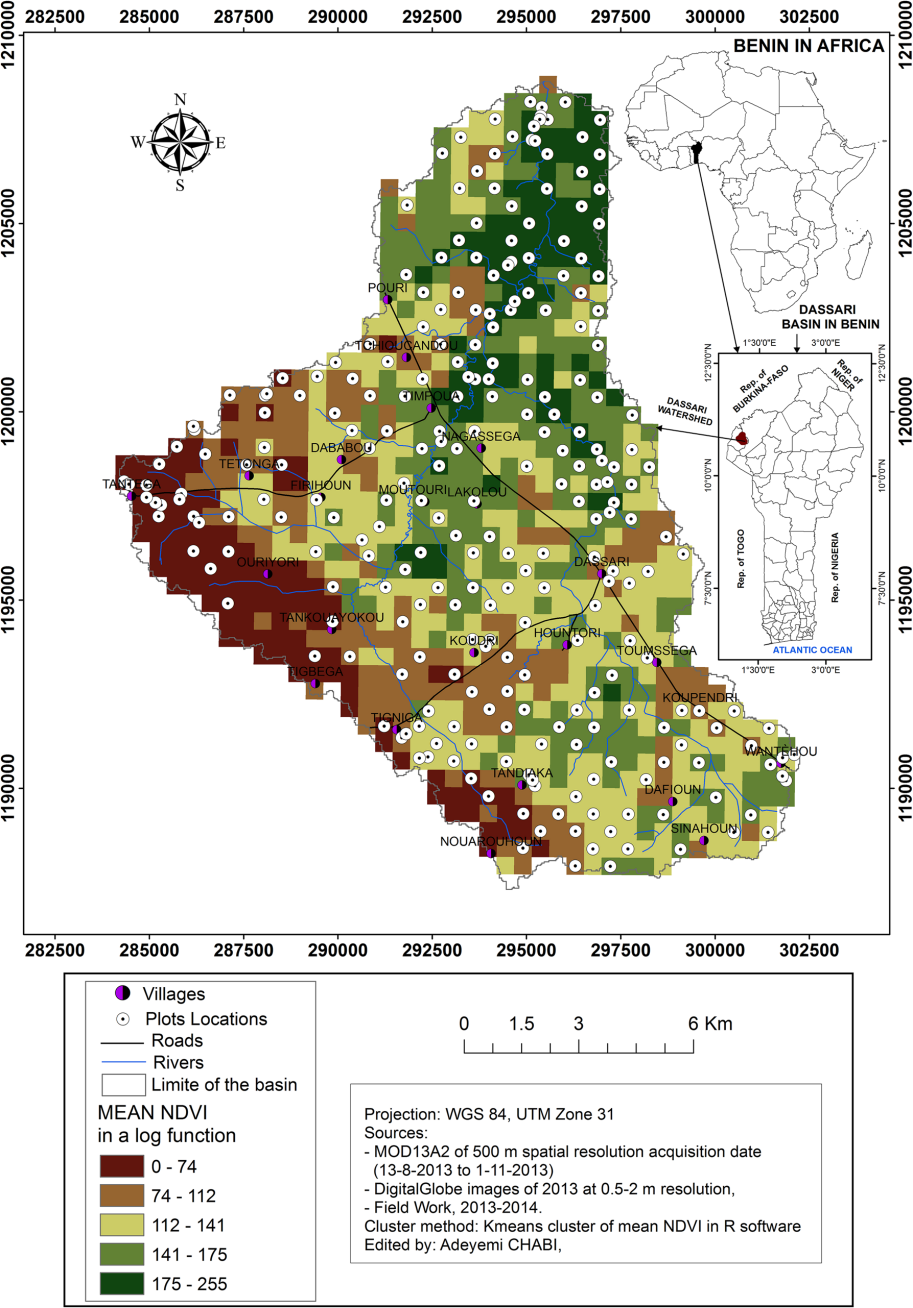
Geographic location of Dassari watershed in North-West of Benin and the gridded vegetation index map with plots locations

The analysis took place in the Dassari basin situated in North-West of Benin (Fig. 2) and covers an area of 192.57 km^2^. The site is located between 10°44′08″ and 10°55′42″ North and 1°01′32″ and 1°11′30″ East.

Long-term (1952–2010) minimal daily temperature at Natitingou station located 50 km from the site ranged from 15.25 to 25.08 °C with an average of 20.53 °C. Daily maximum temperature ranged from 26.63 to 39.27 °C with a mean temperature of 32.59 °C. Long-term (1971–2013) mean monthly precipitation for Tanguieta station (15–20 km from the study area) was 87.5 mm.

We used the standardized precipitation index (SPI) programme developed by Brown [68] and the result showed two periods (1978–1979; 1985–1986) of extreme drought with some years of moderate to severe drought during these 42 years of observation.

### Data collection

#### Data sources for images classification

The estimation of aboveground tree biomass at the watershed scale was complicated by the heterogeneous tree species distribution across the different LUCa. Two Landsat 8 scenes (http://glovis.usgs.gov) were used for LULC classification. The acquisition dates were 13 October 2013 and 29 October 2013 both with path-row 193-53. The acquisition dates were chosen since they fit well with the high photosynthetic activity of natural vegetation, crops and offset cloud cover and fire pattern disturbance. The scenes selected had zero percent cloud cover. Landsat 8 images were provided atmospherically and geometrically corrected (Landsat 8, level 2A product).

To separate agroforestry and plantation which are easily confused with natural vegetation at the 30 m Landsat resolution we used Worldview-2 imagery^1^ (0.46/1.84 m resolution panchromatic/multispectral) from with additional ground truthing data (field surveys of plots data from agroforestry systems and plantations).

#### Establishment of gridded vegetation index map using MODIS data

To select sample points that cover the different land use classes adequately, we first derived clusters of land use based on time series of the moderate-resolution imaging spectro-radiometer (MODIS) normalized difference vegetation index (NDVI) product. These clusters were then used as strata in a stratified sampling procedure to select sampling points.

The NDVI [87] is one of the most widely used vegetation indexes and is correlated with several biophysical properties of the vegetation canopy, such as leaf area index, fractional vegetation cover, vegetation condition, and biomass. The NDVI is defined base on the relationship between near infrared (NIR) and red light (RED) and relates the difference of both wave lengths to their sum (Eq. 1). It is built on the observation that chlorophylls a and b in green leaves strongly absorb light in the Red while the cell walls strongly scatter light in the NIR region [88]. NDVI normalizes values between −1 and +1; dense vegetation has a high NDVI, while soil values are low but positive, and water is negative due to its strong absorption of NIR.

We used MODIS data with 500 m resolution and 0 % cloud cover from August 2013 to November 2013 (https://lpdaac.usgs.gov/products/modis_products_table). We calculated the mean NDVI (Eq. 2) of six times series per pixel across time. The mean NDVI was used as input in a k-mean cluster analysis [89] with the number of clusters set to the number of LUCa (forest land, grassland, cropland and settlements and other land use) used in the analysis. The clusters were then used for a stratified random sample creation in ArcGIS 10.2.1. The centroids of the selected pixels were used to establish plots at which ground training area information was derived for the classification (Figs. 1, 2).

1 $$ {\text{NDVI}}_{i} = \frac{{{\text{NIR}}_{i} - {\text{Red}}_{i} }}{{{\text{NIR}}_{i} + {\text{Red}}_{i} }} $$

2 $$ {\text{Mean}}_{{{\text{NDV}}I_{i} }} = \frac{{\mathop \sum \nolimits_{i = 1}^{N} \left( {NDVI_{{\left( {x,y} \right)}} i} \right)}}{N} $$

3 $$ \begin{aligned} & {\text{standardized}}\,{\text{Mean}}_{{{\text{NDVI}}_{\text{i}} }} \\ & \quad = \left[ {\left( {{\text{Mean}}_{{{\text{NDVI}}_{\text{i}} }} - {\text{NDVI}}_{ \hbox{min} } } \right)/\left( {NDVI_{max} - NDVI_{min} } \right)} \right] \times \left( {2^{8} - 1} \right)  \\ \end{aligned} $$

where NDVI = normalized difference vegetation index, NIR = near-infrared band of MODIS, Red = red band of MODIS, i = pixel position (i.e. pixel i) in the scene, N = number of scenes or elements, x = longitude coordinate of pixel i, y = latitude coordinate of pixel i, Mean_NDVI_ (i) = mean NDVI of pixel i, Min = minimum value of Mean NDVI of all pixels, Max = maximum value of Mean NDVI of all pixels.

We rescaled NDVI from range [−1; 1] to [0; 255] using the logarithmic function (Eq. 3) in ERDAS Imagine 10 to avoid negative values in data manipulation and visualization (Fig. 2).

Both sample points for calibration as well as for validation were sampled using the same procedure. Validation sample points were not used for the training of the classifier.

#### Image classification

We used seven LULC classes that reflect the dominant land use classes for biomass stocks assessment in our case study region: riparian forest and woodland, Savanna Woodland, shrub savanna, cropland and fallow, settlements, agroforestry and plantation. At some locations in the text we refer to forest land that incorporates the land use category (LUCa) riparian forest and woodland, Savanna Woodland and shrub savanna. We further separated agroforestry and plantation from cropland since an increase of agroforestry and plantation could be a mitigation strategy to climate change.

Based on the ground truthing data derived for the sample points, a random forest classifier was trained and used to classify the Landsat 8 data. For the classification bands 2, 3 and 4 were used. The random forest approach is a machine-learning approach that builds on classification and regression trees but overcomes their sensitivity towards noise in the data. Instead of relying on a single decision tree, using the majority vote of a forest of decision trees fit to bootstrap samples from the original data. While individual decision trees suffer from a high variance of estimates the averaging across the bootstrap sample leads to a significant variance reduction [90, 91]. In addition to bagging approaches, random forests decorrelate the trees by using only a random sample of the variables (i.e. spectral bands) for each split. The analysis was done in R [69] using the package randomForest [70]. Random forest classifiers have been applied successfully in a number of remote sensing studies [92–94] which showed that the approach is superior to the widely used maximum likelihood classifier.

Since it was not possible to separate agroforestry and plantations from forest land at the scale of the Landsat 8 data these classes were separated based on several high resolution Worldview-2 images, known plantation and agroforestry sites and their geometric properties (regular spacing between trees) in ArcGIS 10.2.1. Areas that were identified as either agroforestry or plantation were validated in the field and assigned to the proper class based on the field validation. For the final LULC map identified agroforestry and plantations were superimposed on the existing classification (Fig. 1).

#### Accuracy assessment of the classification

The accuracy of the random forest classification (without the superimposed classification of agroforestry and plantations) was based on independent validation points that were sampled similar to the training sample points. By comparing classification results and observed land use classes at the location of the validation points a confusion matrix was derived. Based on the confusion matrix overall accuracy and the kappa index [71] were derived to assess the accuracy of the classification.

#### Forest inventory

Forest inventory and tree species community analysis was carried out during 7 months (from March to September 2014). In every LULC class, plots were installed randomly proportionally to their size (Table 1) using the equation from Pearson et al. [72]. The size of plots was 30 × 30 m in forest land, grassland and cropland, 100 m × 100 m within the settlements and 10 m × 20 m in agroforestry and plantation. The total 250 plots (Fig. 2; Table 1) have been surveyed which cover 27.26 ha.

**Table 1:** Land use/land cover (LULC) classes and number of installed plots

Characteristics	IPCC [95] land use categories (LUCa)							
	Forestland			Grassland	Cropland	Settlement	Others land use	
LULC	RFW	SW	SS	GL	CPF	SL	AGF	PLT
Area (ha)	341.19	5476.5	4282.56	96.57	8044.47	488.34	20.7	17.1
Percentage in the watershed	1.77	28.44	22.24	0.50	41.77	2.54	0.11	0.09
Area sampled (ha)	0.81	2.43	5.04	3.06	7.2	8	0.26	0.46
Number of establishing plots	09	27	56	34	80	08	13	23

Agroforestry and plantation were seen as mitigation strategies to climate change, we therefore choose to discriminate them from cropland

*RFW* riparian forest and woodland, *SW* Savanna woodland, *SS* shrub Savanna, *GL* grassland, *CPF* cropland and fallow, *SL* settlement, *AGF* agroforestry, *PLT* plantation

#### Importance value index (IVI) analysis

The objective of IVI calculation was the selection of the main species of the watershed for the development of tree biomass allometric equations. The IVI analysis has been used for the first time by Curtis [73] to determine the overall importance of tree species for a tree community structure. The IVI of a species is the sum of the relative frequency, relative density and relative dominance of the species in a region.

Relative density (RD%) for species i:

4 $$ RD_{i} = \frac{Density\, of\,species\,Ai}{Total\,density\,for\,all\,species } \times 100 $$

Relative frequency (RF%) for species i:

5 $$ RF_{i} = \frac{Frequency\,value\,for\,species\,Ai}{Total\,frequency  \,values\,for\,all\,species} \times 100 $$

Relative dominance (RDom%) for species i:

6 $$ RDom_{i} = \frac{Dominance \,for\,species\,Ai}{Total\,dominance\,for\,all\,species } \times 100 $$

Importance value index (IVI):

7 $$ IVI(A_{i} ) = RD_{i} + RF_{i} + RDom_{i} $$

where IVI (A*i*) = importance value index of species A*i* with *i* varied from 1 to N (here N = 81 species), RD_i_ = relative density of species i (%), RF_i_ = relative frequency of species i (%), RDom_i_ = relative dominance of species i (%), Total density for all species = sum of density across all species, Total frequency values for all species = Sum of frequency across all species, Total dominance for all species = sum of dominance across all species.

Among the 84 inventoried species within the entire watershed, only three were not taken into the account for IVI calculation. The first species was *Adansonia digitata* which has DBH range 9.2–185 cm, with a relative abundance of 9.87 % and the density of 0.54 (<1 plant ha^−1^). *Phoenix reclinata* and *Borassus flabellifer* were removed since we could rely on published allometric equations for coconut biomass estimation, by Schoroth et al. [74] for these two species. For the remaining 81 species the IVI was calculated using Eqs. 4–7 to obtain 15 most important tree species. These 15 tree species were used for the further analysis.

#### Field campaign and the estimation of wood density of the main species of the watershed

The materials used for this study were an increment borer, scale weight of 25 kg, metric tape scaling, metre increment and an oven for drying the wood samples. During the second field campaign (from October 2014 to December 2014) wood samples from 270 trees within the 15 main species (*Terminalia macroptera*, *Acacia seyal*, *Combretum glutinosum*, *Pterocarpus erinaceus*, *Anogeisus leiocarpus*, *Mitragyna inermis*, *Lannea microcrapa*, *Lannea acida*, *Ficus* sp., *Crosopteryx febrifuga*, *Entada africana*, *Parkia biglobosa*, *Vitelaria paradoxa*, *Azadirachta indica*, *Anacardium occidentale*) were extracted with the increment borer at 1.3 m above the ground. *A. occidentale* was surveyed in the agroforestry system (cashew). The basic wood density of the samples was estimated after oven-drying them at 75 °C—over 2–3 days depending on the water content of the wood samples.

#### Tree measurements (destructive and non-destructive methods)

It was possible to analyse trees selected for logging in a rural electrification project along the road from Dassari-Tigniga (Fig. 2) in the Dassari basin. Seven species (*T. macroptera*, *Ficus* sp., *A. seyal*, *Entanda Africana*, *C. glutinosum*, *C. febrifuga* and *A. leiocarpus*) and 13 individual trees were selected. Only tree species which were going to be logged by rural electrification project officers and that belonged to the previously mentioned 15 main species (Table 3) of the watershed were analysed. These samples allowed the estimation of the parameters of the BEF function as well as an assessment of the uncertainties attached.

**Fig. 3:**
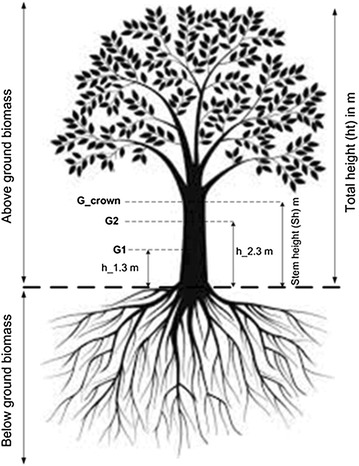
Tree design showing details of measurement of diameters and heights on individual sample tree. Where G_1_ = stem girth of tree at breast height (at 1.3 m), G_2_ = stem girth of tree at 2.3 m height, G_crown = stem girth of tree at the crown base, h_1.3 m = stem height at 1.3 m, h_2.3 m = stem height at 2.3 m

The following activities were undertaken in preparation for the use of the non-destructive approach:

1. Measurement of stem girth at 1.3 m, 2.3 m and crown base, and stem height (Fig. 3);
2. Extraction of stem wood sample of the tree at 1.3 m above ground using the increment borer;
3. Oven-drying the wood sample obtained with the increment borer and estimation of the wood density of the surveyed tree;
4. Estimation of stem-dry mass of the tree species using Eqs. 8–11.

The destructive approach consisted of the following steps:

1. Logging of the tree species by rural electrification project officers,
2. Weighting of fresh mass of stem, branches and foliage using scale weighting of 25 kg,
3. Oven-drying of fresh wood samples selected from stem, branches and foliage at 75 °C for 2–3 days to constant weight;
4. Estimation of dry mass of stem, branches and foliage of the tree using Eq. 12,
5. Calculation of BEF based on dry mass of stem, branches and foliage using Eq. 13,
6. Modelling BEF as a function of stem dry mass using Eq. 14,
7. Comparison of the non-destructive method to the destructive method based on predictive total biomass by BEF function.

Figure 3 shows the various properties collected from sample trees in this watershed.

**Fig. 4:**
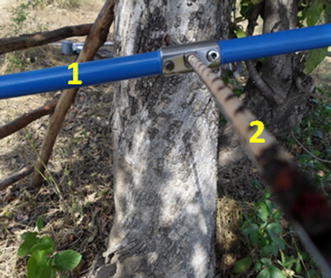
Wood sample obtained from increment borer. CHABI, October 2014. *1* Increment borer, *2* wood sample

The pole of tree height measurement was 4 m in length. One worker holds the pole next to the tree when measuring the stem height. The second worker stood far enough away to estimate the stem height of the tree. Samples of wood were extracted from the tree using an increment borer (Fig. 4).

#### Collection of wood samples in the field

The inner diameter of the bit of the increment borer device was 0.5 cm leading to a diameter of the sample of 0.5 cm. The length L of the sample was measured after its extraction. The application of the tool is shown in Fig. 4.

The main activities preformed related to the destructive approach were the estimation of dry mass of wood samples of stem, branches and foliage followed by the estimation of BEF.

### Data analysis

#### Estimation of basic wood density

The core wood sample was oven-dried at 75 °C during 2–3 days till the stabilization of dry mass. The oven dry density (ρ) in terms of dry mass per fresh volume (g cm^−3^) of all collected wood samples was estimated using Eq. 8.

8 $$ \uprho = \frac{{4{\text{dMS}}_{\text{i}} }}{{\pi d^{2} L_{i} }} $$

where: dMS_i_ = dry mass of wood sample i obtained by the increment borer, d = diameter of the bit, L_i_ = length of the sample i.

#### Estimation of stem volume and stem biomass of surveyed trees

The stem volume of the trees was measured by section according to Fig. 3. The truncated cone function was used to estimate this stem volume (Eq. 9):

9 $$ Vistem_{i} = {\text{H}}_{\text{i}} \times \frac{1}{12\pi } \times \left( { C_{1i}^{2} + C_{2i}^{2} + C_{1i} \cdot C_{2i} } \right) $$

where H_i_ = height in m of section i of the tree stem, C_1i_ = the greater girth of the section i of the tree stem, C_2i_ = the smaller girth of the section i of tree stem, Vistem_i_ = Volume in cm^3^ of section i of the tree stem.

The stem biomass was estimated based on wood density and stem volume values of the sections of the tree stem (Eq. 10) and added up to get the total stem biomass based on non-destructive approach (Eq. 11):

10 $$ Bistem_{i} = \left( {\rho \times Vistem_{i} } \right)/1000 $$

11 $$ {\text{TBstem}} = \mathop \sum \limits_{i = 1}^{n} Bistem_{i} $$

where Vistem_i_ = volume in cm^3^ of section i of the tree stem, Bistem_i_ = biomass in kg of section i of the tree stem, TBstem = total biomass of tree stem, n = number of the section of the tree stem.

In the next step, dry mass of stem, branches and foliage were added up to get the total biomass of the tree based on destructive approach:

12 $$ Btot = \mathop \sum \limits_{j = 1}^{m} \frac{{fM_{j} \times dMS_{j} }}{{fMS_{j} }} $$

where Btot = total biomass of a tree (sum of dry mass of stem, branches and foliage) in kg, fM = fresh mass of stem, branches or foliage, dMS = dry mass of wood sample of stem branches or foliage, fMS = fresh mass of sample of stem branches or foliage, j = index of the different components (stem, branches and foliages), m = number of components, 3 in the present case.

The BEF per tree was calculated using Eq. 13.

13 $$ BEF = \frac{\text{Btot}}{\text{Bstem}} $$

where Btot = total biomass of a tree (sum of dry mass of stem, branches and foliage) in kg, Bstem = stem dry biomass in kg, BEF = biomass expansion factor.

#### Modelling BEF as a function of stem dry mass

The relationship between BEF and stem dry biomass (Bstem) was modelled by a linear regression model in R [75]. Stem dry biomass has been log-transformed to provide a more even spread of the data.

14 $$ {\text{BEF}} = \beta_{0} + \beta_{1} { \ln }(Bstem) + \varepsilon $$

where β_0_ and β_1_ are regression coefficients and ε the error term which we assume to be normally distributed and centred on zero.

#### Fitting aboveground biomass (AGB) equations for the surveyed individual tree species

Total samples consisted of 270 individual trees that have been non-destructively surveyed (Table 3). For each tree of that sample, the BEF was applied to calculate the AGB. This AGB was then modelled by generalized linear models (GLM) [76] using predictors easily measured in the field. We selected DBH, total height (H) and wood density (ρ) as predictors. Since the effort to measure the predictors increases from DBH to H and to ρ we fitted models for three sets of predictors: (1) just on DBH, (2) DBH and H, (3) all three predictors together. Based on the properties of the residuals we decided on a Gamma GLM with a log link. For each level of complexity we started with a model that contained the interactions between all involved predictors as well as the main effects (conditional on the interactions). We tried to simplify the model structure based on the small sample size of corrected Aikaike information criteria (AICc) [77, 78]. Quadratic effects were not considered since their inclusion led to unrealistic model behaviour for higher response values which we interpreted as a result of overfitting the model.

The aim was the fitting of model at land use categories (LUCa) level—i.e. data were subsetted by LUCa before fitting—in addition to a generic category which included all LUCas. Effects of species on the model fit as well as on the structure of the residuals were tested but effects were small. We used the following LUCa to fit the models: forest land (the combination of riparian forest, Savanna Woodland and shrub savanna), savanna grassland (grassland), settlement, cropland (cropland and fallow). The sample size differed by LUCa: agroforestry: 25, forest: 181, cropland: 178, settlements: 63, grassland: 90. We did not fit models for the LUCa plantation but applied published equations. AGB from plots plantations of *Tectona grandis* and *Eucalyptus grandis* were obtained using respectively published allometric equations from Guendehou et al. [42] and Montagu et al. [79] whereas the generic equation (cf. Fig. 7; Additional file 2) was applied to estimate AGB of *A. indica* and *Gmelina arborea.*

#### Validity domain of equations for DBH

The models were run under certain ranges of DBH obtained from each LUCa. The DBH ranges were 5.6–44.7, 7.6–40.7, 6.9–62.4, 7–52.7 and 9.2–57.9 cm respectively in forest land, savanna grassland, cropland and fallow, settlement and agroforestry systems (cashew plantation).

#### Estimating aboveground biomass at the watershed level

We generated the biomass density map using the best specific equation for each LUCa especially equation type III which involved the three predictors. For agroforestry model II was used since wood density did not have a significant effect on AGB estimates in this LUCa. We estimated biomass content of each plot in two steps when we found *P. reclinata* and *B. flabellifer* in the plot data. We first retrieved these species from each plot data and we estimated their biomass using equation from Schoroth et al. [74] developed for the estimation of AGB of coconut. In the second step we applied specific equations for the concerned plots and we summed up together the two results to obtain the total biomass of the plot. The total biomass stocks of each LULC class is equal to the mean AGB density expressed in Mg ha^−1^ times the area in ha of the defined LULC class. The biomass stocks map was edited using ArcGIS 10.2.1 software.

## Results and discussion

### Land use classification

**Fig. 5:**
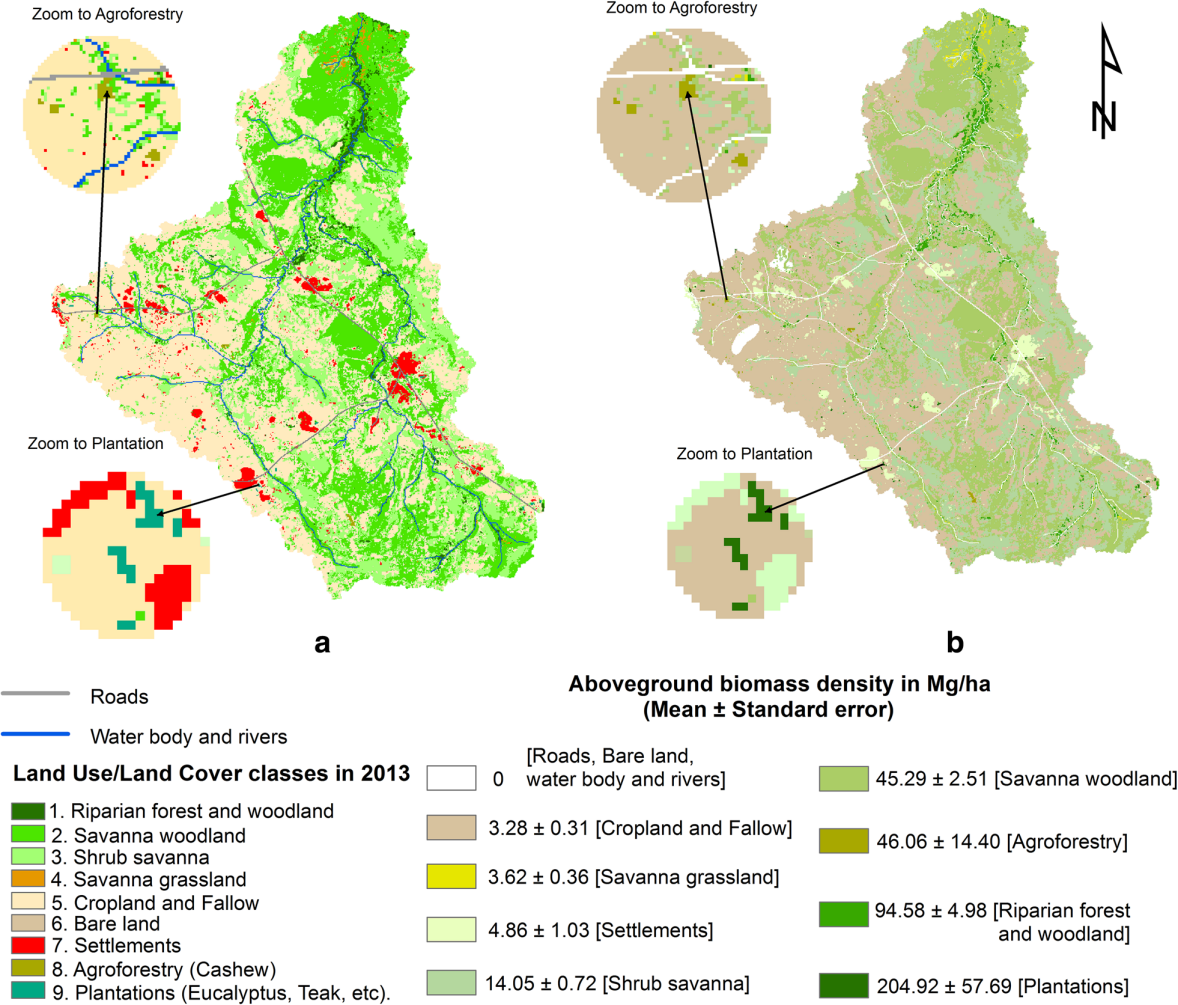
**a** The land use/land cover classes and **b** biomass density of the watershed. The biomass density was expressed in Mg ha^−1^. The total biomass stocks in each LULC are presented in the Table 6

The accuracy of the classification of the Landsat 8 imagery (Fig. 5) was acceptable to good given the overall accuracy of 0.75 and the kappa index of 0.70. Since the identification of the agroforestry systems and plantations was done manually we could not derive an accuracy measure.

### Main species in the study area

Based on the IVI analysis we identified 15 main species that were used for the further analysis. These represented 80.5, 82.75, 79.55 and 76.8 % of the total number of tree species for forest land, grassland, cropland and fallow, and settlement respectively (cf. Table 2).

**Table 2:** Importance value index of main species in each land use/cover category (LUCa)

Species name	Importance value index (IVI) (%)			
	Forestland	Grassland	Cropland	Settlement
*T. macroptera*	42.57	125.85	19.23	–
*A. seyal*	33.18	24.39	21.01	–
*C. glutinosum*	31.13	–	5.99	–
*P. erinaceus*	25.55	–	6.02	–
*A. leiocarpus*	24.09	–	–	–
*M. inermis*	18.22	–	–	–
*L. microcrapa*	16.06	–	44.97	28.25
*Ficus* sp.	8.79	32.28	28.84	42.08
*C. febrifuga*	8.01	–	–	–
*E. africana*	7.32	22.27	–	–
*P. biglobosa*	–	42.14	65.50	–
*V. paradoxa*	–	–	21.28	–
*A. indica*	–	–	15.92	96.63

### Basic wood density of the main species of the study area

Table 3 shows the estimated basic wood density of the main species in the study area. The species *A. leiocarpus*, *C. glutinosum*, *T. macroptera*, *V. paradoxa*, *P. erinaceus*, *A. indica*, *A. seyal*, and *C. febrifuga* were characterized by a high mean wood density. The low mean density observed for *L. microcrapa* and *Ficus* sp. is in line with the high water content of the species which is lost during the drying process. Standard deviations of the measurements were very low for all species and confirmed thereby the accuracy of the measurements as well as the relative low importance of confounding factors which influence density variation per species as described by Chave et al. [56]. Measurements on basic wood density were in line with results from previous studies [57–62].

**Table 3:** The basic wood density (g cm^−3^) of the main species of the watershed

Trees species	The present study						Previous studies *ρ* (g cm^−3^)
	n	Basic wood density		Mean (SE)	DBH (cm)		
		Min.	Max.		Min.	Max.	
*T. macroptera*	19	0.740	0.893	0.821 (0.010)	9.3	40.7	0.768^a^; 0.870^b^
*A. seyal*	16	0.669	0.909	0.751 (0.015)	7.6	34.4	–
*C. glutinosum*	11	0.827	0.962	0.877 (0.013)	7.9	31.9	0.900^b^
*P. erinaceus*	21	0.671	0.973	0.826 (0.015)	6.9	44.7	0.740^a^
*A. leiocarpus*	16	0.813	0.977	0.889 (0.012)	6.9	32.4	–
*M. inermis*	18	0.579	0.687	0.631 (0.008)	7.0	34.5	–
*L. microcrapa*	22	0.472	0.648	0.546 (0.011)	7.0	50.6	–
*L. acida*	06	0.504	0.676	0.573 (0.027)	10.8	35.9	–
*Ficus* sp.	21	0.440	0.607	0.528 (0.010)	8.6	52.7	–
*C. febrifuga*	18	0.518	0.778	0.704 (0.016)	5.6	30.5	–
*E. africana*	15	0.556	0.688	0.631 (0.010)	8.4	27.6	–
*P. biglobosa*	23	0.566	0.689	0.630 (0.006)	8.6	62.4	0.525^c^
*V. paradoxa*	23	0.608	0.950	0.838 (0.016)	8.0	53.8	–
*A. indica*	16	0.619	0.886	0.763 (0.018)	8.8	50.5	0.660^d^; 0.620^e^
*A. occidentale*	25	0.512	0.625	0.569 (0.006)	9.2	57.9	0.431^c^; 0.500^e^

n = Number of tree selected. The stem wood samples of selected trees were extracted at 1.3 m of the ground. DBH range = range of diameter at breast height of sampled tree species. Figures in bracket represent the standard error (SE) of the mean

Authors of previous studies: ^a^ Sallenave [57, 58], ^b^ Von Maydell [59], ^c^ Carsan et al. [60], ^d^ Oey et al. [61], ^e^ Little et al. [62]

### BEF

**Fig. 6:**
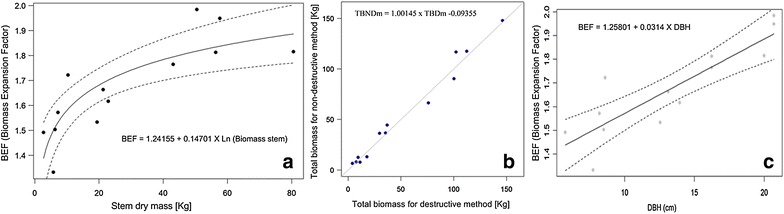
**a** Estimated relationship between stem dry biomass and biomass expansion factor modelled for the 13 trees that were available for the analysis by the destructive approach (*left panel*). The *dashed lines* represent the 95 % confidence band. **b** Comparison between total biomass derived by the destructive and the non-destructive method (*centre panel*). The *grey line* represents the 1:1 line to aid interpretation. **c** Estimated relationships between DBH and biomass expansion factor modelled for the 13 trees that were available for the analysis by the destructive approach (*right panel*). The *dashed lines* represent the 95 % confidence band

The BEF increased significantly with the log of stem dry biomass (Table 4; Fig. 6, left panel). The BEF as a function of stem dry biomass varied between 1.46 and 1.88 with a mean of 1.67 ± 0.04 (SE). The BEF of *T. macroptera* which is the main species of the study site ranged from 1.55 to 1.88 with a mean of 1.73 ± 0.009 (SE). The model explained 69 % of the variance in the data. Residuals showed no relevant pattern with respect to the different tree species.

**Table 4:** Coefficients for the BEF–stem dry biomass relationship and for the BEF–DBH relationship fitted

	Coefficient	SE	p value
BEF–stem dry biomass relationship			
Intercept	1.24155	0.09253	3.66 × 10^−8^
ln(stem dry biomass)	0.14701	0.02968	0.000434
BEF–DBH relationship			
Intercept	1.25801	0.07697	4.61 × 10^−9^
DBH	0.0314	0.00543	0.000122

Both regressions were fitted based on the 13 trees available for the destructive method and non-destructive assessment

Total biomass obtained by the destructive method (observed values from 13 trees) and the total biomasses estimated by the non-destructive method (predicted values from these trees using the estimated BEF–stem dry mass relationship) were highly similar (Fig. 6 centre panel, Pearson correlation coefficient of 0.99).

Our results differ significantly from relationships identified in other land systems stressing the importance of deriving BEF relationships adjusted to the conditions in the ecozone. Segura [63] used a similar approach based on an estimated BEF function for the per-humid premontane transitional forest zone in Costa Rica. In contrast to our findings BEF decreased with stem biomass in Costa Rica. While the Costa Rican study underestimated total biomass of trees on average by 17.31 %, the application of the BEF to our data overestimated total biomass slightly by 1.82 %. Levy et al. [64] estimated the biomass expansion of coniferous species in Great Britain as a function of tree height of stand tree. An application of Levy’s BEF to our data overestimated the total biomass of our sampled tree species to on average by 4.46 %. Magalhães and Seifert [65] used BEF as a function of DBH when estimating AGB of *Androstachysjohnsonii Prain* in Mozambique. The application of the BEF of Magalhães and Seifert [65] to our data underestimated the total biomass of our sampled tree species on average by 62.54 %.

Given the small sample size (13 individual trees within seven species) and the limited range of DBH (<25 cm) care should be taken not to extrapolate results. However, the sampled trees represent the common size distribution of trees in the human influenced ecosystems of the study region. Therefore, our results can be assumed to provide a good estimate for BEF assessments in the region.

Alternatively, BEF could be estimated based on DBH of the 13 trees assessed by the destructive approach (Fig. 6, right panel).The model based on DBH was slightly superior to the model based on stem dry biomass if compared by means of the small sample size corrected AIC (AICc) or a likelihood ratio test and it explained 75 % of the variance in the BEF (Table 4).

If this model was used to predict total biomass, the values derived by the destructive approach were overestimated on average by 2.27 %—a bit higher compared to the model based on stem dry mass. We therefore stuck to the estimation based on stem dry biomass.

### Aboveground biomass (AGB) models at the watershed level

**Fig. 7:**
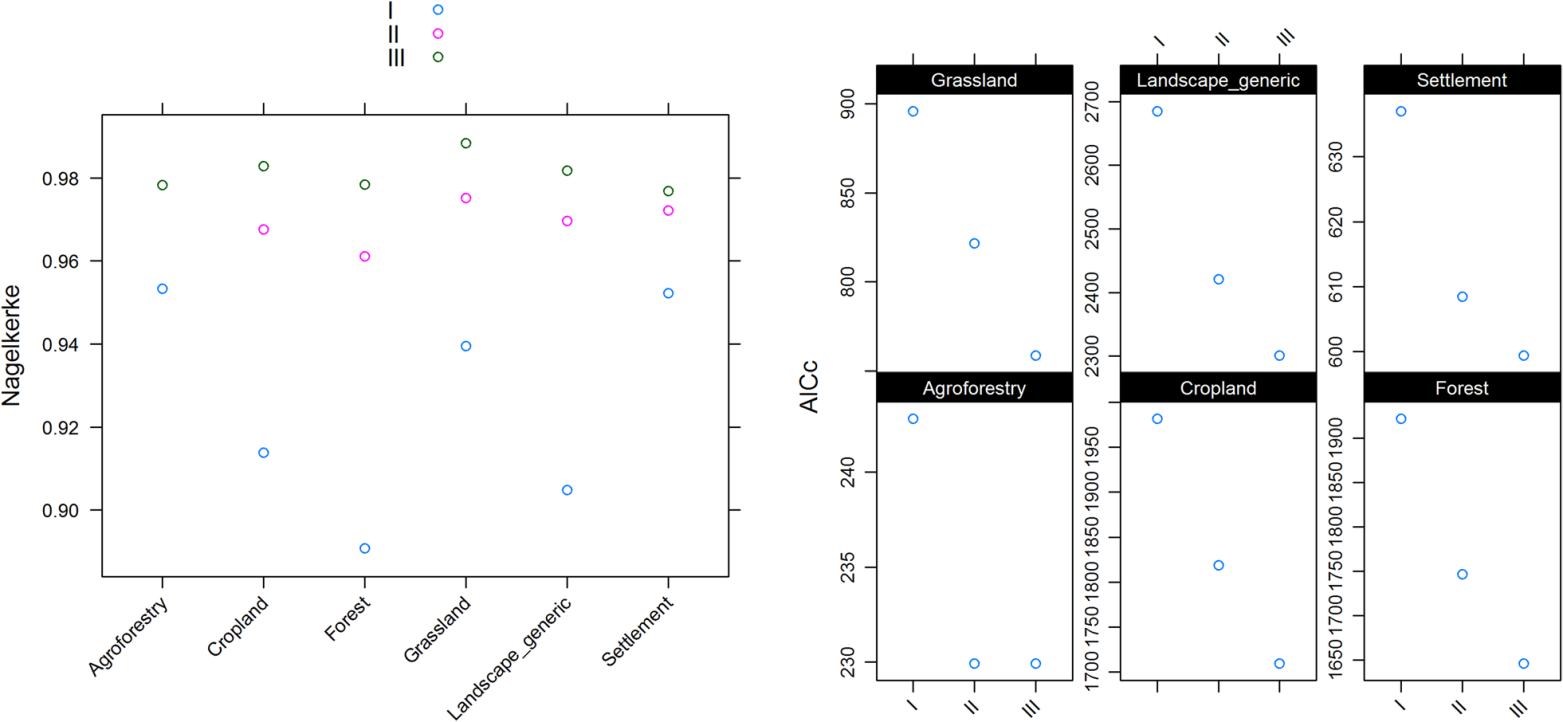
Goodness of fit assessment for the allometric models by land use class for each of the three levels of model complexity analysed. The *left panel* shows the Nagelkerke pseudo-R^2^ while the *right panel* shows the AICc. Please not that AICc values cannot be compared across land use classes and that a delta AICc of >2 indicates that the models clearly differ in respect to their likelihood

**Fig. 8:**
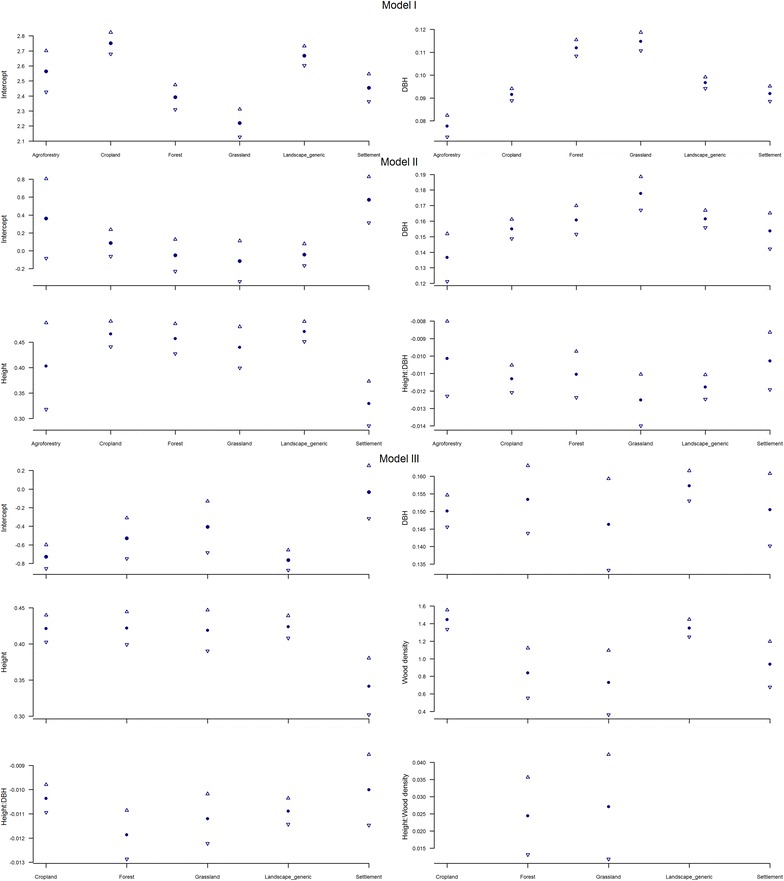
Regression coefficients of the allometric models by land use class for each of the three levels of model complexity analysed. The *filled circle* represents the estimate of the coefficient the *triangles* indicate the standard error of the estimate. For the effect plots please see the figures in the Additional file 1. The data were fitted for the generic model (all watershed) and for each land-use category (LUCa). The coefficients are provided at the link scale. The log-link was used for fitting the gamma glm. The ‘:’ operator represents the interaction between both involved variables. The sample size differed by land use category: agroforestry: 25, forest: 181, cropland: 178, settlements: 63, grassland: 90. Aboveground biomass is based on dry weight (kg tree^−1^). For agroforestry model type III was reduced to the same model structure as model type II—i.e. wood density had no significant effect on the estimation of above ground biomass here. See Additional file 2 for the details

All models indicated a high goodness of fit expressed by the explained deviance as well as by the pseudo-R^2^ by Nagelkerke [66]. While the AICc clearly favoured the more complex models (cf. Fig. 7 even the models using only DBH as a predictor provided a high goodness of fit. An analysis of the effects of LUCa as an additional predictor on all sample points indicated significant differences between the coefficients across LUCa. This is also visible when comparing regression coefficients per model type across LUCa (cf. Fig. 8). Expectedly, the generic landscape model could not capture this variability. However, inside a model class, coefficients always were of same sign and of the same order of magnitude. For models of type II, the inclusion of the interaction between DBH and total height were always selected based on the lower AICc. For the other categories wood density was included in the models in addition to the other two main effects and the interaction between DBH and total height. For forest land and grassland, the interaction between DBH and wood density was also selected based on AICc. The basic wood density (ρ) was not a good predictor for the estimation of AGB in agroforestry system (cashew plantation). This can be explained by the fact that cashew was the only tree species in that LUCa and that wood density measurements for that species had a relatively low variance. In cashew plantations big trees—i.e. cashew trees older than 45 years—tend to lose their wood ignition followed by the observed decrease of wood density for bigger cashew trees. In the available 25 cashew trees wood density was high for cashew trees with an age of 10–20 years.

For model type I regression coefficients for DBH differ by around 40 % with lowest estimates for agroforestry and highest values for forests and grasslands. For model II agroforestry and settlements have clearly distinct coefficient estimates from the other land use classes. Model coefficients for model type III are hard to compare since the interaction between wood density and height was only significant for forest and grasslands. Effects plots (cf. Additional file 1) indicated, that the differences between regression coefficients across the LUCa led to important changes in prediction.

### Comparing the equations to previously published equations

We could only compare our allometric models for forest lands with previous published allometric equations due to the lack of allometric equations for cropland, grassland and settlements. We therefore compared only the models for forest land and the generic model with results from Brown [67], Chave et al. [22], Chave et al. [3] and de Jose [45]. We choose equations developed by these authors in the global dry forest region for the comparison. Our results were in line with all mentioned equations in terms of mean deviation of the observed AGB at the stand tree level (Table 5). Our model type I which was only based on DBH differed on average by 21.67 % from the observed AGB for forest land. Results from the equations based on DBH for Brown [67] and de Jose [45] differed on average by 25.02 and 26.41 % respectively. The same analysis was done with the model type II and III in comparison with the previous studies when DBH was not the only predictor. Model type III differed on average by 4.77 % for forest land whereas Chave et al. [22] and Chave et al. [3] respectively differed by 9.04 and 14.78 %. This highlights the importance of allometric models adjusted to the regional conditions in the Sudan Savannah ecosystems in West Africa.

**Table 5:** The average deviation of various models compared to the models type of the present study in each LUCa

LUCa	Previous studies				Present study		
	Brown [67]	Chave et al. [22]	José [45]	Chave et al. [3]	Models type		
					I	II	III
	Average deviation δ (%)						
Forest land	25.02	9.04	26.41	14.87	21.67	8.64	4.77
Grassland	34.23	6.54	35.73	12.69	11.88	5.00	2.34
Cropland	29.30	10.21	30.77	14.74	24.50	10.06	5.26
Agroforestry	–	–	–	–	8.00	3.75	–
Settlements	60.93	9.70	62.77	15.46	12.40	7.30	6.35
Generic	–	9.00	–	14.19	–	–	5.34

### Aboveground biomass density and stocks at the watershed level

The results for biomass density in Savanna Woodland and riparian forest were in the same magnitude with those obtained by Sidzabda et al. [80] in forest land of the Sudan Savannah zone of Burkina-Faso. The mean biomass density and attached standard error varied from 3.28 ± 0.31 to 204.92 ± 57.69 Mg ha^−1^ across the LUCas (Fig. 5; Table 6) with the lowest biomass density in cropland and the highest biomass density in plantations emphasizing the potential of plantations as a mitigation strategy for the climate change. Biomass density differed however strongly between the different trees used in plantations. High uncertainty of biomass estimates for plantation and agroforestry system might be due to the different age of plots data; since biomass increases with stand age [81]. Since our LULC data classification could not separate between young and old cashew tree plantations we have unfortunately to deal with this high uncertainty. This highlights the need for additional data to assess the potential of agroforestry and plantations as a mitigation strategy to climate change.

**Table 6:** Aboveground biomass density (Mg ha^−1^) and total biomass stocks (Mg) with the sample plots data and attached uncertainty

LULC/LUCa	Descriptive statistic				
	Range of biomass density (Mg ha^−1^)		Mean biomass density (SE)	Percentage error (% error)	Total biomass stocks (Mg) and its SE
	Min.	Max.			
Forest land					*340,534.70* ± *36,445.4*
Riparian forest and woodland	76.29	120.22	94.58 (4.98)	(10.33)	32,271.87 ± 334.74
Savanna Woodland	27.22	69.84	45.29 (2.51)	(10.89)	24,8050.22 ± 27,019.98
Shrub Savanna	6.47	25.14	14.05 (0.72)	(10.11)	60,212.61 ± 6090.67
Grassland					*349.66* ± *68.81*
Savanna grassland	0.06	9.20	3.62 (0.36)	(19.68)	349.66 ± 68.81
Cropland					*26,409.82* ± *5024.04*
Cropland and fallow	0.07	9.32	3.28 (0.31)	(19.02)	26,409.82 ± 5024.04
Settlements					*2375.84* ± *988.13*
Settlements	0.86	9.60	4.86 (1.03)	(41.59)	2375.84 ± 988.13
Agroforestry					*1132.73* ± *584.46*
Cashew plantation	10.74	211.19	46.06 (14.40)	(61.28)	1132.73 ± 584.46
Plantation					*3138.20* ± *1777.35*
*E. grandis*	7.69	695.20	204.92 (57.69)	(55.17)	2819.78 ± 1556.44
*T. grandis*	32.41	232.75	162.00 (64.88)	(78.50)	145.80 ± 114.46
*A. indica*	64.45	240.53	179.62 (57.61)	(62.86)	129.33 ± 81.30
*G. arborea*	10.39	34.39	25.17 (7.46)	(58.09)	43.29 ± 25.14

Words in italic indicate the plantation type

Value within the parenthesis indicate the standard error and percentage error

The age of plantations and agroforestry system varied from 5 to 45 years which explained the large standard and percentage errors obtained from their plots data. The minimum (min.) and maximum (max.), the mean biomass density and its stand error (SE), the percent error and the total biomass at each LULC type/LUCa were illustrated

The biomass density map (Fig. 5) was generated based on the best model for each LUCa. The map in Fig. 5 shows the LULC types and the biomass density at the watershed level specifically for each LULC class. Information on the uncertainties of the biomass density estimation is provided in Table 6.

## Conclusion

The results from this study help to close the existing knowledge gap with respect to biomass estimation in the Sudan Savannah environment. The derived empiric equations fitted to local data should be useful for further work in the Sudan Savannah environment which is characterized by the main species of the present study. The estimation of biomass density and AGB in each LUCa are of great importance for carbon balance calculations in the Sudan Savannah in West Africa. Results include data on wood density of the main species of the Sudan Savannah zone, the related BEF and the biomass density in each LUCa. Our results highlight the importance of model parameters adjusted to the regional conditions in the eco-zone. Plantations and agroforestry system could be a useful mitigation option to battle climate change—however, the differences between the species and the effect of age which could not be satisfyingly handled in our study call for additional research activities. Still, the results provide important information for the carbon accounting programme related to the implementation of the Kyoto Protocol and REDD+ initiatives.

## Additional files

**Additional file 1.** Effect plots for the allometric models for each LUCa.

**Additional file 2.** Parameters and expressions of the allometric models generated using diameter at breast height DBH (cm), height H (m) and wood density ρ (g cm^−3^).

## Abbreviations

BEF: biomass expansion factor
DBH: diameter at breast height
AGB: aboveground biomass
LULC: land use/land cover
LUCa: land use category

## Footnotes

1 Worldview-2 imagery can be found at http://www.digitalglobe.com.
