# Erratum to: Allometric models and aboveground biomass stocks of a West African Sudan Savannah watershed in Benin

**DOI:** 10.1186/s13021-016-0064-7

**Published:** 2016-09-29

**Authors:** Adéyèmi Chabi, Sven Lautenbach, Vincent Oladokoun Agnila Orekan, Nicholas Kyei‑Baffour

**Affiliations:** 1 Department of Civil Engineering, Kwame Nkrumah University of Science and Technology, Kumasi, Ghana; 2 Faculty of Agriculture, Institute of Geodesy and Geo‑Information, University of Bonn, Nussallee 1, 53115 Bonn, Germany; 3 Department of Geography, University of Abomey-Calavi, BP 677, Abomey-Calavi, Benin; 4 Department of Agricultural Engineering, The College of Engineering, Kwame Nkrumah University of Science and Technology, Kumasi, Ghana; 5 West African Science Service Centre on Climate Change and Adapted Land Use (WASCAL), Accra, Ghana

## Erratum to: Carbon Balance Manage (2016) 11:16 DOI 10.1186/s13021-016-0058-5

Upon publication of the original article [1], the authors noticed an error in the legend for Table 6. Instead of:

“Worlds in italic indicate the LUCa type”

This should read:

“Words in italic indicate the plantation type”

This has now been updated in the original article.
