# Supplementary material for: Allometric models and aboveground biomass stocks of a West African Sudan Savannah watershed in Benin
Source: Carbon Balance Manag. 2016 Aug 17;11:16. doi: 10.1186/s13021-016-0058-5 (PMC4989002; doi:10.1186/s13021-016-0058-5)
Supplement: Supplementary file 1 — Additional file 1. Effect plots for the allometric models for each LUCa. [file 13021_2016_58_MOESM1_ESM.pdf]

Appendix – Effect plots for allometric models

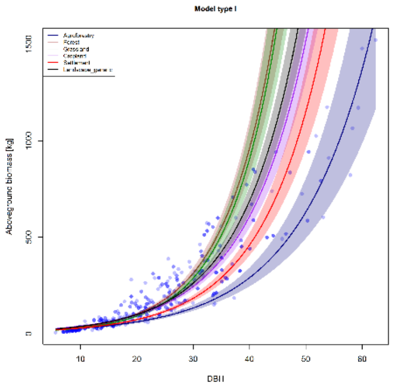

Figure 1. Effect plots of model type I. The points represent the measurement, the bold lines the fitted curve for the land use class specific model and the shaded areas represent the 95% confidence bands.

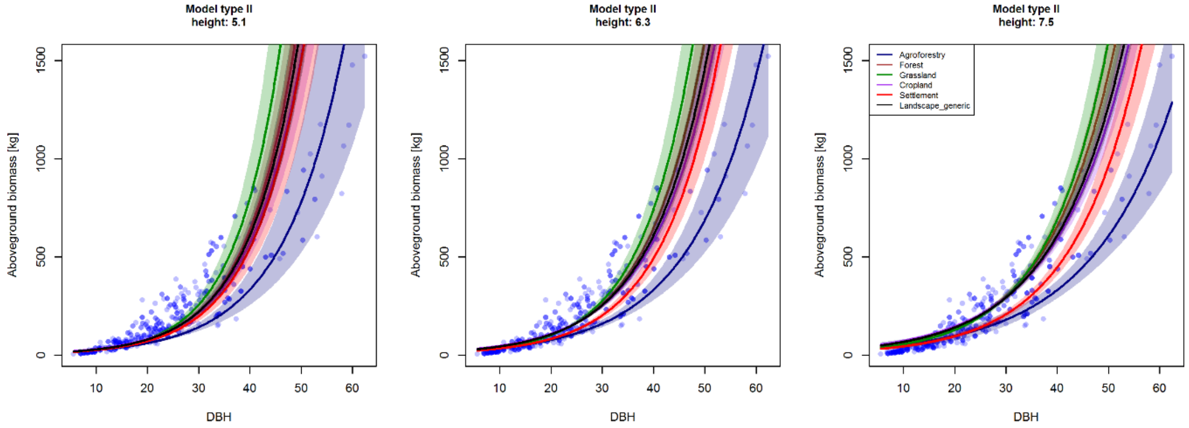

Figure 2. Effect plots of model type II. The points represent the measurement, the bold lines the fitted curve for the land use class specific model and the shaded areas represent the 95% confidence bands. The tree height has been set to the mean (middle row) or the 25- or 75-percentile from the data.

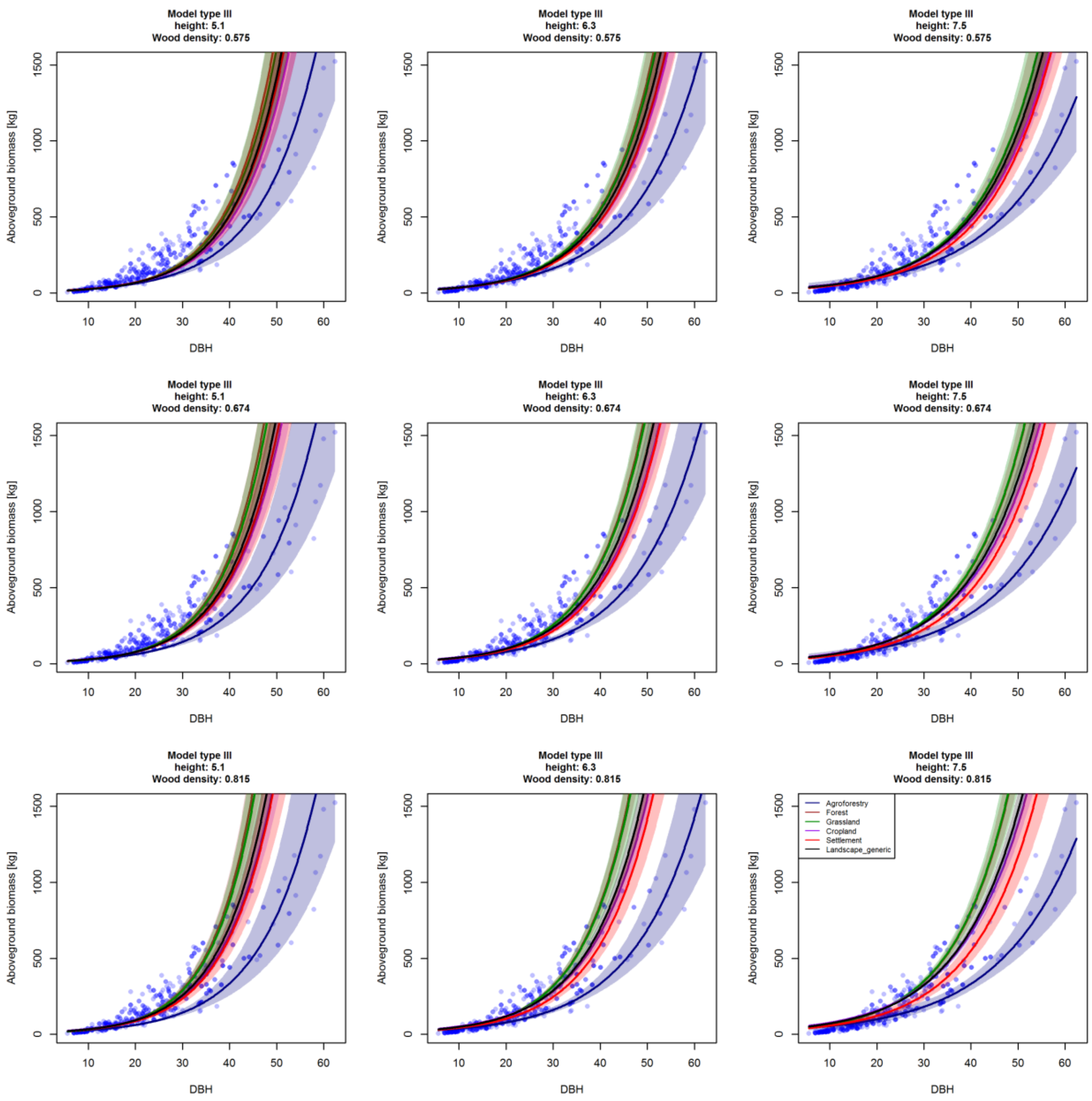

Figure 3. Effect plots of model type III. The points represent the measurement, the bold lines the fitted curve for the land use class specific model and the shaded areas represent the 95% confidence bands. The tree height has been set to the mean (middle row) or the 25- or 75-percentile from the data. Wood density has been set accordingly to the 25-percentile, the mean and the 75-percentile (first, second and third column).
