# Supplementary material for: Allometric models and aboveground biomass stocks of a West African Sudan Savannah watershed in Benin
Source: Carbon Balance Manag. 2016 Aug 17;11:16. doi: 10.1186/s13021-016-0058-5 (PMC4989002; doi:10.1186/s13021-016-0058-5)
Supplement: Supplementary file 2 — Additional file 2. Parameters and expressions of the allometric models generated using diameter at breast height DBH (cm), height H (m) and wood density ρ (g cm−3). [file 13021_2016_58_MOESM2_ESM.pdf]

Appendix 2. Parameters and expressions of the allometric models generated using diameter at breast height DBH (cm), height H (m) and wood density ρ ($g.cm^{-3}$)

| Models | LUCa | Intercept | DBH | H | DBH:H | ρ | DBH:ρ | AIC | Expl. Dev. | Nagelkerke |
|---|---|---|---|---|---|---|---|---|---|---|
| | **Coefficients** | **$\beta_0$** | **$\beta_1$** | **$\beta_2$** | **$\beta_3$** | **$\beta_4$** | **$\beta_5$** | | | |
| **I** | $\boldsymbol{ln\,(AGB)}$ = | $\boldsymbol{\beta_0}$ + | $\boldsymbol{\beta_1(DBH)}$ | | | | | | | |
| | Forest land | 2.391980*** | 0.111911*** | | | | | 1921.3 | 0.82 | 0.89 |
| | | (0.082228) | (0.003528) | | | | | | | |
| | Grassland | 2.219779*** | 0.114745*** | | | | | 895.75 | 0.90 | 0.94 |
| | | (0.092797) | (0.004002) | | | | | | | |
| | Cropland | 2.751514*** | 0.091492*** | | | | | 1981.3 | 0.84 | 0.91 |
| | | (0.072229) | (0.002608) | | | | | | | |
| | Settlements | 2.454958*** | 0.091898*** | | | | | 636.64 | 0.91 | 0.95 |
| | | (0.091445) | (0.003292) | | | | | | | |
| | Agroforestry | 2.563685*** | 0.077676*** | | | | | 241.67 | 0.92 | 0.95 |
| | | (0.137175) | (0.004729) | | | | | | | |
| **II** | $\boldsymbol{ln\,(AGB)}$ = | $\boldsymbol{\beta_0}$ + | $\boldsymbol{\beta_1(DBH)}$ + | $\boldsymbol{\beta_2(H)}$ + | $\boldsymbol{\beta_3(DBHxH)}$ | | | | | |
| | Forest land | -0.051323 | 0.160755*** | 0.456829*** | -0.011051*** | | | 1745.9 | 0.93 | 0.96 |
| | | (0.178402) | (0.009200) | (0.029571) | (0.001321) | | | | | |
| | Grassland | -0.115578 | 0.177817*** | 0.439903*** | -0.012521*** | | | 821.11 | 0.96 | 0.98 |
| | | (0.227236) | (0.010732) | (0.040451) | (0.001474) | | | | | |
| | Cropland | 0.0871685 | 0.1549490*** | 0.4660558*** | -0.0113066*** | | | 1818.4 | 0.94 | 0.97 |
| | | (0.1495242) | (0.0062096) | (0.0251691) | (0.0007811) | | | | | |
| | Settlements | 0.570740* | 0.153706*** | 0.329399*** | -0.010279*** | | | 607.38 | 0.95 | 0.97 |
| | | (0.257908) | (0.011535) | (0.043811) | (0.001639) | | | | | |
| | Agroforestry | 0.361587 | 0.136600*** | 0.403086*** | -0.010145*** | | | 226.77 | 0.96 | 0.98 |
| | | (0.444254) | (0.015331) | (0.085069) | (0.002141) | | | | | |
| **III** | $\boldsymbol{ln\,(AGB)}$ = | $\boldsymbol{\beta_0}$ + | $\boldsymbol{\beta_1(DBH)}$ + | $\boldsymbol{\beta_2(H)}$ + | $\boldsymbol{\beta_3(DBHxH)}$ + | $\boldsymbol{\beta_4(\rho)}$ + | $\boldsymbol{\beta_5(DBHx\rho)}$ | | | |
| | Generic | -0.7654108*** | 0.1573235*** | 0.4238142*** | -0.0108973*** | 1.3500342*** | | 2300.2 | | |
| | | (0.1091666) | (0.0042834) | (0.0155108) | (0.0005404) | (0.1004703) | | | | |
| | Agroforestry | Model reduced to the type II model | | | | | | | | |
| | Forest land | -0.529352* | 0.153447*** | 0.421777*** | -0.011862*** | 0.838169** | 0.024398* | 1645.1 | 0.96 | 0.98 |
| | | (0.218806) | (0.009621) | (0.022671) | (0.001007) | (0.285044) | (0.011265) | | | |
| | Grassland | -0.406853 | 0.146300*** | 0.418648*** | -0.011198*** | 0.729644* | 0.027054° | 757.26 | 0.98 | 0.99 |
| | | (0.276970) | (0.013038) | (0.028276) | (0.001026) | (0.366277) | (0.015229) | | | |
| | Cropland | -0.7272044*** | 0.1501417*** | 0.4212572 *** | -0.0103647*** | 1.4462214*** | | 1709.1 | 0.97 | 0.98 |
| | | (0.1278645) | (0.0045440) | (0.0185620) | (0.0005729) | (0.1095952) | | | | |
| | Settlements | -0.031603 | 0.150500*** | 0.341267*** | -0.010006*** | 0.938432*** | | 597.9 | 0.96 | 0.98 |
| | | (0.284948) | (0.010299) | (0.039210) | (0.001463) | (0.260039) | | | | |

**Note:** The data were fitted for the generic model (all watershed) and for each land-use category (LUCa). The coefficients are provided at the link scale. The log-link was used for fitting the gamma glm. The '**:**' operator represents the interaction between both involved variables. Standard error is provided in parenthesis. The sample size differed by land use category: agroforestry: 25, forest: 181, cropland: 178, settlements: 63, grassland: 90. AGB = Aboveground biomass based on dry weight (kg/tree).The statistical analyses are significant at 95% confidence interval. ***p < 0.001; **p < 0.01; *p < 0.05; and non-significant, °p > 0.05.
